# Identification of fungal causative agents of rhinosinusitis from Mashhad, Iran 

**DOI:** 10.29252/cmm.3.3.5

**Published:** 2017-09

**Authors:** Mohammad. J Najafzadeh, Karim Jalaeian Samani, Jos Houbraken, Majid Alizadeh, Abdolmajid Fata, Ali Naseri, Hossein Zarrinfar, Mehdi Bakhshaee

**Affiliations:** 1 Department of Parasitology and Mycology, Faculty of Medicine, Mashhad University of Medical Sciences, Mashhad, Iran; 2 Cancer Molecular Pathology Research Center, Mashhad University of Medical Sciences, Mashhad, Iran; 3 Department of Otorhinolaryngology Head and Neck Surgery, Ghaem Hospital, Mashhad University of Medical Sciences, Mashhad, Iran; 4 Westerdijk Fungal Biodiversity Institute, Utrecht, the Netherlands; 5 Allergy research center, Mashhad University of Medical Sciences, Mashhad, Iran; 6 Sinus and Surgical Endoscopic Research Center, Faculty of Medicine, Mashhad University of Medical Sciences, Mashhad, Iran

**Keywords:** Calmodulin, Identification, ITS, Fungal rhinosinusitis, Molecular technique

## Abstract

**Background and Purpose:**

Rhinosinusitis is a common disorder, influencing approximately 20% of the population at some time of their lives. It was recognized and reported with expanding recurrence over the past two decades worldwide. Undoubtedly, correct diagnosis of fungi in patients with fungal rhinosinusitis affects the treatment planning and prognosis of the patients. Identification of the causative agents using the standard mycological procedures remains difficult and time-consuming.

**Materials and Methods:**

Based on clinical and radiological parameters, 106 patients suspected of fungal rhinosinusitis were investigated in this cross-sectional prospective study from April 2012 to March 2016 at an otorhinolaryngology department. In this study, internal transcribed spacer (ITS) and calmodulin (*CaM*) sequencing were respectively validated as reliable techniques for the identification of Mucorales and *Aspergillus* to species level (both agents of fungal rhinosinusitis).

**Results:**

Of these, 63 (59.4%) patients were suspected of allergic fungal rhinosinusitis (AFRS), 40 (37.7%) patients suspected of acute invasive fungal rhinosinusitis (AIFRS), and 3 (2.8%) patients suspected of mycetoma. In patients suspected of AFRS, AIFRS, and mycetoma only 7, 29, and 1 had positive fungal culture, respectively. After ITS and *CaM* sequencing, *Aspergillus flavus* was the most common species isolated from non-invasive forms, and *A. flavus *and *Rhizopus oryzae* were more frequently isolated from invasive forms.

**Conclusion:**

*A* *spergillus flavus* is the most common agent of fungal rhinosinusitis in Iran, unlike most other reports from throughout the world stating that *A. fumigatus* is the most frequent causative agent of this disease.

## Introduction

Fungi are considered to be major etiological agents of rhinosinusitis, especially members of the genus *Aspergillus* and the order Mucorales that are the most commonly reported agents of fungal rhinosinusitis [1, 2]. Rhinosinusitis is a common disorder that may affect up to 20% of the population [3, 4]. Fungal rhinosinusitis is globally being revealed and reported with expanding recurrence over the past two decades [5]. The clinical manifestation and duration of the illness depend on host’s immune status and vary from innocuous and slight presentation to alive threatening complications [6]. Because of the growing prevalence of chemotherapy, human immunodeficiency virus (HIV) infections, and diabetes mellitus, the number of susceptible immune-compromised hosts is expanding [7].

Fungal rhinosinusitis can be categorized as invasive or noninvasive according to histopathological findings such as fungal invasion to tissue [8-10]. Acute invasive fungal rhinosinusitis occurs in severely immune-compromised patients in a period of less than four weeks, and in all of them hyphae invasion to tissue are observed [11, 12], whereas chronic invasive and noninvasive forms occur in immunocompetent patients [8, 13]. The invasive fungal rhinosinusitis include (a) acute, (b) granulomatous and (c) chronic forms. The noninvasive diseases include (a) allergic fungal rhinosinusitis and (b) fungal ball [6, 8]. Undoubtedly, the diagnosis of fungi in patients with fungal rhinosinusitis affects the treatment planning and prognosis of infection. Fast diagnosis and precise identification of pathogenic fungal species are essential for successful therapy and medical decision-making [14, 15]. 

Laboratory diagnosis of fungal rhinosinusitis relies on microscopic morphology of the agents and has low specificity. The nuclear ribosomal internal transcribed spacer (ITS) region is the universal DNA barcode marker for fungi, and the calmodulin gene is the recommended barcode for *Aspergillus* identification [16, 17]. In this study, we used ITS-rDNA and *CaM* sequences for rapid and accurate identification of fungal isolates.

## Materials and Methods

***Patients’ samples***

In this prospective cross-sectional study, 106 samples were obtained from patients presenting to the Department of Otorhinolaryngology, Mashhad University of Medical Sciences, Mashhad, Iran, with suspected fungal rhinosinusitis over a period of 48 months (April 2012 to March 2016). This study was approved by the Ethics Committee of Mashhad University of Medical Sciences, Mashhad, Iran (Ethical code: IR.MUMS.REC.1392.95). 

Surgically excised specimens were sent in sterile normal saline to a mycology laboratory. Minced tissue specimens were examined directly after mounting with 10% KOH and Geimsa staining. Some portions of the positive tissue samples were inoculated on Sabouraud Dextrose Agar (Merck, Germany) plate containing chloramphenicol (50 mg/mL) and incubated for seven days at 37°C. In positive cultures, the isolate was primarily identified by morphology of the colonies and microscopic morphology by Lactophenol Cotton Blue dye and slide culture technique [18].

***DNA extraction***

DNA extraction was performed using Genet Bio kit (Genet Bio, Korea) with some modifications. Approximately, 1 cm^2^ of 3 to 7 day-old cultures was transferred to a 2-mL Eppendorf Tube containing 400 mL of TEX buffer (pH 9.0) and glass beads (Sigma G9143, SigmaeAldrich, Steinheim, Germany). The fungal material was vortex homogenized for 1 min. The following steps were according to Genet Bio Kit.

***DNA amplification and sequencing***

The ITS region and/or a part of the calmodulin (*CaM*) gene were chosen for the identification of the strains. PCR was conducted in 25 µL of reaction mixture, comprising 7 µL Master Mix containing MgCl_2_, dNTPs, 1 µL of every primer (10 pmol), reaction buffer and 1 µL of gDNA. Amplification was done in an ABI PRISM 2720 (Applied Biosystems, Foster City, USA) thermocycler using the primers ITS1 (5′ CCGTAGGTGAACCTGCGG 3′) and ITS4 (5′ TCCTCCGCTTATTGATATGC 3′) for ITS and primers CMD5 (5′ CCG AGT ACA AGG ARG CCT TC3′) and CMD6 (5′ CCG ATR GAG GTC ATR ACG TGG 3′) for calmodulin. The following conditions were applied: 95°C for 4 min, followed by 35 cycles consisting of 95°C for 45 s, 52°C for 30 s, and 72°C for 2 min, and a final extension at 72°C for 7 min. Annealing temperature was changed to 55°C for the *CaM* gene. Concentrations of amplicons were estimated on gel, photographed, and analyzed by the Gel Doc XR system (Biorad, USA), with SmartLadder (Eurogentec, Seraing, Belgium) as the size and concentration marker. Amplicons were purified using GFX PCR DNA and Gel Band Purification Kit (GE Healthcare, Ltd., Buckinghamshire UK). Amplicons were subjected to direct sequencing as follows: 95°C for 1 min followed by 30 cycles consisting of 95°C for 10 s, 50°C for 5 s, and 60°C for 2 min. Sequencing was performed using ABI PRISM BigDye TM Terminator Cycle Sequencing Kit (Applied Biosystems, Foster City, USA), and sequences were analyzed on an ABI PRISM 3730XL Sequencer. Sequences were edited using SEQMAN in the Lasergene software (DNASTAR, Wisconsin, USA). Etiological agents were identified by comparison of the generated sequences in Genbank using the BLAST search program.

## Results

The findings are based on the study of 106 (60 [56.6 %] males and 46 [43.4 %] females) patients undergone functional endoscopic sinus surgery (FESS) with the mean age of 41.5 years (range: 15 to 83 years). Of these, 63 (59.4%) patients were suspected of allergic fungal rhinosinusitis (AFRS), 40 (37.7%) patients suspected of acute invasive fungal rhinosinusitis (AIFRS), and 3 (2.8%) patients suspected of fungus ball. In patients suspected of AFRS, AIFRS, and fungus ball, only 7, 29, and 1 had positive fungal culture, respectively.

In AFRS, 43 (68%) patients had seasonal allergies, 13 (21%) patients had permanent allergies, and 7 patients did not have any allergies. The most common clinical symptoms among the patients were nasal congestion (95%), nasal discharge (77%), hyposmia (74%), facial pain (71%), anosmia (19%), and headache (8 %), and 60 (95%) patients had eosinophilia.

The most common underlying disease in AIFRS patients were leukemia (52.5%) and diabetes (47.5%) and the most common signs were facial numbness (67.5%), nasal congestion (42.5%), plate numbness (27.5%), diplopia (15%), facial pain, and visual loss (5%) and blindness (5%).

In AFRS patients, direct microscopic examination showed that 46 (73%) out of 63 samples contained branched septate hyphae and 17 (27%) samples were negative. In direct microscopy, just 7 (11%) samples had positive culture. The agents identified by direct microscopy were *Aspergillus fumigatus* (n=3) and *A. flavus* (n=4). With the sequencing method, two of the samples which were initially identified as *A*.* fumigatus* were re-identified as *A. flavus*. *A. flavus *was the most common organism isolated in six patients followed by *A. fumigatus *in one patient (Table 1).

In AIFRS patients, direct microscopic examination showed that 18 (45%) samples contained branched septate hyphae, 16 (40%) samples contained non-septate hyphae, and 6 (15%) samples were negative. In direct microscopy, 29 (72.5%) samples had positive culture. The results for 25 of the 29 samples that were sequenced were in agreement with the results of direct microscopy. Three of these samples in direct microscopy were identified as *A. fumigatus*, but re-identified as *A. flavus*, and one sample in direct microscopy was identified as *A. niger and *re-identified as *A. tubingensis* by sequencing.

*A. flavus* and *Rhizopus oryzae* were the most commonly identified fungi in 13 and 12 patients, respectively, followed by *A. fumigatus* in three patients and *A. tubingensis* in one patient (Table 1).

**Figure 1:**
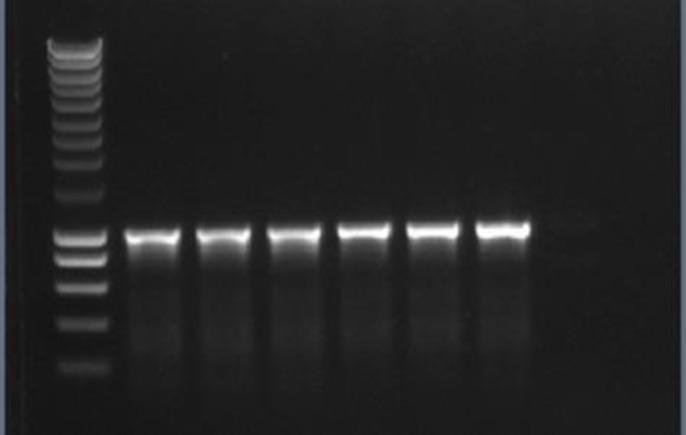
Agarose gel electrophoresis of rDNA ITS products from the tested strains

In one of the three patients suspected of fungus ball, direct microscopic examination showed branched septate hyphae, and by direct microscopy and sequencing it was identified as *A. flavus*. PCR amplification of ITS rDNA and *CaM* yielded products of about 550 bp (Figure 1). 

The new sequences determined in this study were deposited in the NCBI GenBank database with the accession numbers MF685299 to MF685322 for rDNA ITS and MG490645 to MG490648 and MG601231 to MG601240 for *CaM*.

**Table 1:** Comparison of the cultures and sequencing results of fresh samples obtained from patients with allergic fungal rhinosinusitis and acute invasive fungal rhinosinusitis

**No of samples**	**Direct microscopy result**	**Culture**	**Sequencing results of ITS or CaM**	**AFRS**	**AIFRS**
1	Positive	*A. fumigatus*	*A. fumigatus*	Positive	…
2	Positive	*A. fumigatus*	*A. flavus*	Positive	…
3	Positive	*A. fumigatus*	*A. flavus*	Positive	…
4	Positive	*A. flavus*	*A. flavus*	Positive	…
5	Positive	*A. flavus*	*A. flavus*	Positive	…
6	Positive	*A. flavus*	*A. flavus*	Positive	…
7	Positive	*A. flavus*	*A. flavus*	Positive	…
8	Positive	*A. flavus*	*A. flavus*	…	Positive
9	Positive	*A. flavus*	*A. flavus*	…	Positive
10	Positive	*A. flavus*	*A. flavus*	…	Positive
11	Positive	*A. flavus*	*A. flavus*	…	Positive
12	Positive	*A. flavus*	*A. flavus*	…	Positive
13	Positive	*A. flavus*	*A. flavus*	…	Positive
14	Positive	*A. flavus*	*A. flavus*	…	Positive
15	Positive	*A. flavus*	*A. flavus*	…	Positive
16	Positive	*A. flavus*	*A. flavus*	…	Positive
17	Positive	*A. flavus*	*A. flavus*	…	Positive
18	Positive	*A. fumigatus*	*A. flavus*	…	Positive
19	Positive	*A. fumigatus*	*A. flavus*	…	Positive
20	Positive	*A. fumigatus*	*A. flavus*	…	Positive
21	Positive	*A. fumigatus*	*A. fumigatus*	…	Positive
22	Positive	*A. fumigatus*	*A. fumigatus*	…	Positive
23	Positive	*A. fumigatus*	*A. fumigatus*	…	Positive
24	Positive	*A. niger*	*A. tubingensis*	…	Positive
25	Positive	*R.oryzae*	*R. oryzae*	…	Positive
26	Positive	*R.oryzae*	*R. oryzae*	…	Positive
27	Positive	*R.oryzae*	*R. oryzae*	…	Positive
28	Positive	*R.oryzae*	*R. oryzae*	…	Positive
29	Positive	*R.oryzae*	*R. oryzae*	…	Positive
30	Positive	*R.oryzae*	*R. oryzae*	…	Positive
31	Positive	*R.oryzae*	*R. oryzae*	…	Positive
32	Positive	*R.oryzae*	*R. oryzae*	…	Positive
33	Positive	*R.oryzae*	*R. oryzae*	…	Positive
34	Positive	*R.oryzae*	*R. oryzae*	…	Positive
35	Positive	*R.oryzae*	*R. oryzae*	…	Positive
36	Positive	*R.oryzae*	*R. oryzae*	…	Positive

## Discussion

Our recent studies demonstrate the utility of rDNA ITS and *CaM* sequencing for rapid detection and accurate fungal identification. rRNA genes are highly conserved in all fungal species tested to date. PCR amplification and sequencing of the ITS region and a part of the calmodulin gene allows unambiguous identification of Mucorales and *Aspergillus* species, respectively, that are difficult to identify via the classical mycological techniques [17, 19]. Species recognition on the basis of the traditional phenotypic techniques is usually time-consuming and laborious and is restricted by the unpredictable and subjective character of phenotypic features, which are immediately affected by culture conditions. However, molecular techniques involving gene sequencing are objective, produce results which can be uninfluenced by growth situations, are often faster than phenotypic methods, and are designed for differentiating between fungi that fail to create specific morphological characters. 

In our study, results of culture and sequence analysis of patients’ samples were concordant with 86.5% of culture positive cases, and this percentage is slightly higher than that in the study of Willinger et al. [20], where a percentage of 73.3% was observed. In our experience, non-invasive fungal rhinosinusitis was seen in 62% of the patients (AFRS: 59.4% and FB: 2.8%) and was more than invasive FRS (AIFRS: 37.7%). A relatively similar distribution was reported in studies performed in India. Das et al. [21] observed non-invasive FRS in 60% of patients (AFRS: 56% and FB: 4%) and invasive FRS in 36% of patients, Montone et al. [12] in a retrospective study in 400 patients with FRS observed non-invasive FRS in 87.5% of patients and invasive FRS in 36% of patients, and Michael et al. [22] observed a prevalence of 63% for AFSR and 24% for AIFRS. In the USA, Granville et al. [23] and Taxy [3] reported non-invasive disease in over 80% of patients. On the other hand, Challa et al. [24] observed a much lower incidence of non-invasive FRS in south India, and this diversity may be due to different climates and environmental factors.

 In this study the sensitivity of fungal culture in AFRS patients was 15.2% (7 of 46), in another study by Polzehl et al. [25] in Germany, sensitivity of culture was 24.7% (19 of 77), negative culture results could be attributed to the exclusive presence of non-viable fungal elements. FRS has the most variaty of fungi isolated in different geoghraphic areas and diferent studies. In the current study, *A. flavus* is apparently the most frequent fungal organism cultured in AFRS, which was also noted in studies by Al- Dousary [26] in Saudi Arabia and Das et al. [21] in India. In AFRS patients in the USA, especially in the south and southwest, mostly dematiaceous fungi grow in culture [23, 27]. *Rhizopus *sp*ecies*, *A. fumigatus*, and *A. flavus *are the main causes of AIFRS around the world [21, 22]. In our study, *Rhizopus oryzae *and *A. flavus *were the most common fungi isolated from AIFRS patients. In this study, similar to other studies [3, 21, 24], FB was the least common form of FRS, althouth Montone et al.[12] and Panda et al. [11] in India and Dufour et al.[28] in France noted that FB was the most common form of FRS in their patients.

## Conclusion

*A* *spergillus flavus* is the most common agent of fungal rhinosinusitis in Iran, unlike most other reports from throughout the world stating that *A. fumigatus* is the most frequent causative agent of this disease.
